# Solid-Phase Synthesized Copolymers for the Assembly of pH-Sensitive Micelles Suitable for Drug Delivery Applications

**DOI:** 10.3390/nano12111798

**Published:** 2022-05-24

**Authors:** Razvan Ghiarasim, Crina Elena Tiron, Adrian Tiron, Mihail-Gabriel Dimofte, Mariana Pinteala, Alexandru Rotaru

**Affiliations:** 1 Centre of Advanced Research in Bionanoconjugates and Biopolymers, “Petru Poni” Institute of Macromolecular Chemistry, 41A Grigore Ghica Voda Alley, 700487 Iasi, Romania; ghiarasim.razvan@icmpp.ro; 2 TRANSCEND Centre, Regional Institute of Oncology, 2-4 General Henri Mathias Berthelot Street, 700483 Iasi, Romania; transcendctiron@iroiasi.ro (C.E.T.); adrian.tiron@iroiasi.ro (A.T.); mihail.dimofte@umfiasi.ro (M.-G.D.)

**Keywords:** polyhistidine copolymers, micelles, doxorubicin, tumor cells, release studies

## Abstract

Diblock copolymers of polyhistidine are known for their self-assembly into micelles and their pH-dependent disassembly due to the amphiphilic character of the copolymer and the unsaturated imidazole groups that undergo a hydrophobic-to-hydrophilic transition in an acidic pH. This property has been largely utilized for the design of drug delivery systems that target a tumor environment possessing a slightly lower extracellular pH (6.8–7.2). The main purpose of this study was to investigate the possibility of designed poly(ethylene glycol)-polyhistidine sequences synthesized using solid-phase peptide synthesis (SPPS), to self-assemble into micelles, to assess the ability of the corresponding micelles to be loaded with doxorubicin (DOX), and to investigate the drug release profile at pH values similar to a malignant extracellular environment. The designed and assembled free and DOX-loaded micelles were characterized from a physico-chemical point of view, their cytotoxicity was evaluated on a human breast cancer cell line (MDA-MB-231), while the cellular areas where micelles disassembled and released DOX were assessed using immunofluorescence. We concluded that the utilization of SPPS for the synthesis of the polyhistidine diblock copolymers yielded sequences that behaved similarly to the copolymeric sequences synthesized using ring-opening polymerization, while the advantages of SPPS may offer facile tuning of the histidine site or the attachment of a large variety of functional molecules.

## 1. Introduction

For biomedical applications, the pH-sensitive materials or assemblies that are stable at physiological pH (pH = 7.4) and unstable at lower pH (pH = 5.0–7.2) [1,2,3,4,5], together with the efficient carriers of a different nature [6,7,8,9,10], are of great interest [1,2,3,4,5]. Particularly, for drug delivery applications in cancer treatment, these types of materials, if properly tuned, enable the release of therapeutics within tumor tissues (pH = 6.5–7.2) [11,12,13,14,15,16,17], making the pH sensibility an excellent strategy for targeted chemotherapeutics delivery. A large variety of efficient, pH-sensitive drug carriers has been developed and reported in the last two decades [12,13,18,19]. The design of the carrier typically involves the incorporation of acid-labile groups into the copolymer structure [20,21], covalent grafting of the drug molecules to copolymer nanostructures via acid-labile linkers [22], or the presence of a large, pH-sensitive moiety that suffers conformational changes at lower pH values, leading to the disruption of the self-assembled nanostructures [23,24]. The latter approach has been largely utilized in the construction of core–shell micelles involving the self-assembly of amphiphilic block copolymers [1,2,11,25,26]. In this context, polymer–peptide conjugates that undergo pH-dependent structural changes can be used as highly competitive drug carriers that target both the tumor extracellular environment and intracellular compartments, with demonstrated in vitro and in vivo tumor inhibition [24,27,28]. These poly(amino acid)-containing drug carriers possess ionizable side-chain groups, such as imidazole (histidine), amino (lysine), or guanidinium (arginine), that are able to strongly influence the amphiphilicity and, correspondingly, the carrier stability at low pH values. In particular, the poly(histidine) based assemblies received much attention because of their high pH sensibility due to overhanging unsaturated imidazole groups that undergo a hydrophobic-to-hydrophilic transition at an acidic pH due to protonation [29,30]. Moreover, the combination of poly(histidine) with poly(ethylene) glycol (PEG) enhances the assembly’s drug carrier circulation time, improves the drug uptake, and slows renal clearance [30,31,32,33]. In their pioneering works, Bae and co-workers reported the synthesis of PEG poly(histidine) block copolymers with a controlled molecular weight of a cationic polymer using ring-opening polymerization of protected L-histidine N-carboxyanhydride with a corresponding primary or secondary amine initiator [34,35]. The resultant block copolymers were obtained within a narrow molecular weight distribution with an assembly into core–shell-type micelles and showed excellent results as smart drug delivery systems [36,37] or regarding the detection of small tumors in vivo [38]. The developed synthetic approach for the synthesis of PEG poly(histidine) block copolymers was subsequently successfully adapted for the design of a large variety of smart multifunctional nanoparticle systems [11,30,31,39,40]. Despite numerous reports on PEG poly(histidine) micellar systems for loading and releasing anticancer drugs in vitro and in vivo, the major drawback of this strategy represents the complexity of the block copolymers’ building blocks synthesis, which requires special conditions for the ring-opening polymerization reaction. This fact confines the application and testing of these systems by research groups with limited or nonexistent synthetic facilities. This constraint motivated us to find and test readily accessible alternatives to prepare PEG poly(histidine) diblock copolymers without the employment of an organic chemistry infrastructure. A route toward this goal may represent solid-phase peptide synthesis (SPPS) [41,42,43,44], which, due to the development of a wide variety of excellent coupling reagents, resins with better physical properties, novel linkers, and orthogonal protecting groups, permits facile synthesis of virtually any short peptides. With a few exceptions, the synthesis of short peptide sequences (20–50 amino acids) is considered a reasonably simple process [42]. Highly standardized SPPS methods are convenient because they allow for efficient multi-step syntheses on a small or large scale, with rapid and easy workups, and minimal material loss. SPPS is utilized by peptide synthesis companies that offer custom peptides with a flexible range of scales and purities, as well as a diverse spectrum of terminal modifications, dyes, and labels. To the best of our knowledge, the assembly of the pH-sensible micelles using PEG poly(histidine) block copolymers synthesized using SPPS has not yet been reported. Herein, we report the design, preparation, and evaluation of pH-responsive nanoparticle systems based on model PEG poly(histidine) sequences prepared using SPPS, along with drug loading and delivery applications. The designed copolymeric sequences were constructed from a 2 kDa PEG unit linked to the polyhistidine moiety with a variable length (20, 26, and 32 amino acids) containing a terminal lysine unit to ensure covalent binding of amine-reactive fluorophores [45], together with the cysteine unit at the other end of polyhistidine to attach the PEG through a maleimide reaction. The prepared sequences were evaluated for the formation of assemblies, their pH-responsive properties, antitumor drug (doxorubicin: DOX) loading, and in vitro assessment on a human breast cancer cell line (MDA-MB-231), where the results were compared with similar reported systems.

We used DOX in our experimental design based on its wide use in the treatment of different types of cancers, such as ovarian, breast, prostate, and lung [46]. In addition, DOX exhibits auto-fluorescence, which is a useful tool to evaluate the released DOX from loaded micelles in a pH-dependent manner [47].

## 2. Materials and Methods

### 2.1. Materials

mPEG-2000-Mal-Cys-(His)20-Lys (90.01% purity), mPEG-2000-Mal-Cys-(His)26-Lys (90.36% purity), and mPEG-2000-Mal-Cys-(His)32-Lys (90.30% purity) were purchased from Chempeptide Limited (Shanghai, China). Doxorubicin hydrochloride pharmaceutical secondary standard; certified reference material), pyrene (98.00%), sodium phosphate dibasic (Na_2_HPO_4_, ≥99.00%), and potassium phosphate monobasic (KH_2_PO_4_, ≥99.00%) were acquired from Sigma-Aldrich (St. Louis, MO, USA). Dimethyl sulfoxide (≥99.90%) and potassium chloride (≥99.00%) were purchased from Honeywell (Charlotte, NC, USA). Sodium chloride (99.80%) was delivered by VWR International (Radnor, PA, USA). Hydrochloric acid (0.1 N) and sodium hydroxide (0.1 N) were purchased from Supelco (Bellefonte, PA, USA). Ultrapure water was used for 1X phosphate-buffered saline. Drug-loaded micelles were purified using a dialysis membrane (Pre-treated RC Rubing, MWCO–2 kDa, Spectrum Labs, San Francisco, CA, USA). An MDA-MB-23 cell line was purchased from the American Type Culture Collection (Manassas, VA, USA). DMEM-F12 (Biological Industries, Beit HaEmek, Israel), DPBS (Biological Industries), trypsin-EDTA solution (Sigma-Aldrich, St. Louis, MO, USA), fetal bovine serum (American Type Culture Collection, Manassas, VA, USA), penicillin/streptomycin (Biological Industries), and 96-well plates with black flat bottoms (Ibidi, Fitchburg, WI, USA) were also purchased.

### 2.2. Determination of the Critical Micelle Concentration of the Three Copolymers

The critical micelle concentrations of the three studied copolymers were determined via fluorescence using pyrene as the standard chemical compound. Thus, a concentration of 5 mM pyrene in acetone (stock solution) was obtained via dilution to 0.5 μM of pyrene in 1X PBS (pH 7.4) after the evaporation of acetone from 2 μL of the stock solution. For each copolymer, sets of solutions with concentrations between 0.5 mg/mL and 0.0009 mg/mL of the copolymer in 1X PBS (pH 7.4) were prepared, followed by the addition of pyrene with the final concentration of 0.5 μM in each solution. The samples were next subjected to magnetic stirring for 18 h under light-protected conditions with a 2 h resting period, followed by emission spectra recording with the excitation of the pyrene at 334 nm at 25 °C. The ratio of the pyrene fluorescence intensities of the excimer band (505 nm) and the peak 3 band (386 nm) in the emission spectra was determined as a function of the copolymer concentration, after normalization of the spectra at the maximum intensity given by the first peak of the pyrene. These ratios were represented as a logarithmic concentration and the Boltzman sigmoidal equation, where the intersection of the lower tangents of this sigmoid represented the value of the critical micelle concentration of the investigated sample.

### 2.3. Assembly of Copolymers in Unloaded Micelles and DOX-Loaded Micelles

The assembly of copolymers into micelles and then loading micelles with DOX followed an adapted protocol that was reported earlier [32]. Thus, in the case of unloaded micelles, 30 mg of each copolymer was dissolved in 30 mL of aqueous DMSO solution (1:1 DMSO:water, the mixture indicated a pH of 7.3), with a subsequent magnetic stirring (400 rpm) at 25 °C for 4 h to form micelles. Next, the micellar solution was introduced into a dialysis membrane (MWCO 2 kDa) and dialyzed against 1000 mL of 1X PBS (pH 7.4). After 24 h, the PBS was replaced with a fresh one and the dialysis continued for another 24 h. The mixture of DMSO:water was gradually replaced with 1X PBS (pH 7.4) to yield 30 mL of final micellar solution in 1X PBS (pH 7.4) with a concentration of 1 mg copolymer/mL. In the case of DOX-loaded micelles, the same protocol for assembling copolymers in micelles was followed, except that in this case, 5 mg of DOX was added to each copolymer in the initial DMSO:water solution, with the removal of unloaded excess DOX during the dialysis.

### 2.4. Encapsulation Efficiency (EE) and Drug Loading (DL) of DOX

The encapsulation efficiency (EE) and the drug loading (DL) of DOX-loaded micelles were determined by following the emission spectra of DOX. The three samples were analyzed in 1X PBS (pH 7.4) with a dilution factor of 60 of 1 mg copolymer/mL DOX-loaded solutions and the values were compared to the standard DOX calibration curve in 1X PBS at pH 7.4 (Figure S5a). The EE and DL were calculated using the following formulas according to the literature [48]:

$$\mathrm{EE}\;\left(\%\right)=\left(\frac{Weight\;of\;\mathrm{DOX}\;in\;micelles}{Weight\;of\;feeding\;\mathrm{DOX}}\right)\times 100\;\;\mathrm{DL}\;\left(\%\right)=\left(\frac{Weight\;of\;encapsulated\;\mathrm{DOX}\;in\;micelles}{Total\;weight\;of\;micelles}\right)\times 100\;$$

### 2.5. In Vitro pH-Dependent Release Studies

The in vitro release model of doxorubicin from DOX-loaded micelles was performed using the dynamic dialysis method [49]. First, the amount of DOX loaded into the three types of micelles was calculated. Next, all three samples, together with 1 mg free DOX in 1X PBS (pH 7.4), were introduced separately into dialysis membranes (MWCO: 2 kDa) and dialyzed against 300 mL of 1X PBS at pH values of 7.4, 7.2, and 6.5. The DOX amount released from the micelles at a temperature of 37 °C and stirring at 200 rpm was monitored at established intervals (0.5, 1, 2, 4, 6, 8, and 10 h) by taking 3 mL of the sample from the release dialyzed medium, with the subsequent replacement of the taken volume by 3 mL 1X PBS buffer. The samples were quantified using a fluorescence measurement of DOX emission (592 nm). Each sample was analyzed in triplicate and the result was represented as a cumulative DOX release, which is a parameter calculated using the following formula [49]:

$$\mathrm{Cumulative}\;\mathrm{DOX}\;\mathrm{release}\;\left(\%\right)=\frac{W_{t}\times 100}{W}$$

where *W_t_* is the amount of DOX released at time *t* and *W* is the amount of DOX in DOX-loaded micelles.

### 2.6. Characterization

The potentiometric titration curves of the three copolymers were performed with an SI Analytics Titrator TitroLine^®^ 7000 titrator (SI Analytics GmbH, Mainz, Germany) in an aqueous solution in the pH range 2–12, with a copolymer concentration of 1 mg/mL. The solutions were first adjusted to pH = 2.00 using 0.1 M HCl and subsequently titrated with 0.1 M NaOH to pH = 12.0.

The hydrodynamic diameter of the unloaded micelles and DOX-loaded micelles was measured via dynamic light scattering (DLS) using a Delsa Nano C Submicron Particle Size Analyzer (Beckman Coulter, Brea, CA, USA) at a concentration of 1 mg copolymer/mL in 1X PBS at pH 7.4. The samples were measured in triplicate with 70 iterations each and the standard deviations were computed.

Zeta potential values of unloaded micelles and DOX-loaded micelles were measured with a Delsa Nano C Submicron Particle Size Analyzer (Beckman Coulter, Brea, CA, USA) using the corresponding module (Flow Cell Module) at a concentration of 1 mg copolymer/mL in 1X PBS at pH 7.4. Each sample was measured at five points with 10 iterations in triplicate and the standard deviations were subsequently calculated.

The fluorescence spectra were measured using a FluroMax-4 spectrophotometer (Horiba, Kyoto, Japan) for both DOX (excitation at 480 nm, emission with the start at 495 nm and the end at 945 nm, increment 1, and slit set at 3.5 nm) and pyrene (excitation at 334 nm, emission with the start at 349 nm and the end at 653 nm, increment 1, and slit set at 2 nm).

A scanning electron microscope measurements were performed on a Verios G4 UC (Thermo Fisher Scientific, Waltham, MA, USA) in STEM mode at 10.00 kV with STEM 3 + detector (light field mode). Samples were deposited from an aqueous solution at 250 μg copolymer/mL 1X PBS (pH 7.4) on carbon-coated copper grids with 400-mesh, and then the solvent was removed under vacuum. The distribution of the micelles’ diameters for both the unloaded and the DOX-loaded micelles was performed with the ImageJ program [50].

For immunofluorescence investigations, the samples were treated with NuncBlue (Invitrogen, Waltham, MA, USA) for 30 min (nuclear staining) and immunofluorescence imaging was performed using Zeiss Axio Observer Z1 (Carl Zeiss AG, Jena, Germany) from a Tissue Gnostic (Wien, Austria) rig; TissueFaxs 4.2 software (TissueGnostics, Vienna, Austria) was used for image acquisition.

### 2.7. Cell Culture

Triple-negative human breast cancer MDA-MB-231 (ATCC^®^, Rockville, MD, USA), which was a generous gift from James Lorens (Bergen Bio, Bergen, Norway), was used in the experimental design. MDA-MB-231 cells were cultured in F-12K Medium, supplemented with 100 U/mL of penicillin, 100 g/mL of streptomycin, and 5% fetal bovine serum.

### 2.8. Cell Viability

For the cell viability estimation, a CellTiter-Cell Viability Assay (Promega, Madison, WI, USA) was utilized. Cells were seeded into a 96-well flat-bottom tissue culture plate (Ibidi, Gräfelfing, Germany) at a density of 3000 cells/well and allowed to adhere to the plate by incubating overnight at 37 °C in 5% CO_2_. When the pH reached 7.4, the cells were treated with either unloaded or DOX-loaded micelles at 1 µg/mL DOX. After a 72 h treatment period, 50 μL of cell viability solution was added to each well and the plate was re-incubated for 4 h before absorbance recording using a multi-plate reader (FilterMax F5, Sunnyvale, CA, USA).

### 2.9. Fluorescence Measurements

Fluorescent dyes were protected by using ProLong Gold Antifade Mountant with DAPI (ThermoFisher Scientific P36941) according to the manufacturer’s recommendations. Pictures were acquired at 20× using a Zeiss Axio Observer Z1 Microscope from TissueGnostic rig and TissueFAXS 4.2 software. The DOX auto-fluorescence was identified using FITC filters.

### 2.10. Statistical Analysis

GraphPad Prism software was used for the statistical analysis. Grouped analyses were performed using one-way ANOVA. Significance was established when *p* < 0.05.

## 3. Results and Discussion

### 3.1. Synthesis of Copolymers

Three diblock copolymeric sequences (PEG2K-PHis20, PEG2K-PHis26, and PEG2K-PHis32) with a constant length of the PEG (2 kDa) unit and variable poly(histidine) (PHis) moiety were synthesized by Chempeptide Ltd. (Shanghai, China) [51] using SPPS. The binding between PEG and PHis was performed directly on the solid support via the reaction between the thiol group of the cysteine bound to the PHis end and a maleimide functionalized PEG. Additionally, a lysine amino acid was introduced at the end of the PHis part in order to have an available amino group for the possible micelles’ internal labeling involving amino reactive probes. The copolymers were analyzed using mass spectrometry and high-performance liquid chromatography (Figures S1–S3).

The determined pK_a_ values of the sequences ranged from 6.94 for PEG2K-PHis20 to 6.97 for PEG2K-PHis26, with a slightly higher value of 7.01 for PEG2K-PHis32 (Figure S4). These data were in agreement with previously reported pK_a_ values of PEG-PHis systems synthesized using ring-opening polymerization [34,38] and were slightly higher when compared with the polyhistidine homopolymer due to increased hydration of the PEG [52]. The obtained pK_a_ values provided by the amphoteric nature of imidazole of the PEG2K-PHis sequences suggested their pH-sensitive behavior.

### 3.2. Critical Micelle Concentration (CMC)

Since the association of copolymeric PEG2K-PHis sequences into micelles is a function of concentration, the determination of CMC using fluorescence of pyrene was utilized [34]. The CMC was determined in 1X PBS buffer (pH 7.4) due to the enhanced stability of micelles at this pH value and the importance of the obtained data at this pH for both in vitro and in vivo applications. Figure S5 shows the emission spectra of the pyrene and mixture of pyrene with different concentrations of PEG2K-PHis sequences. The emission spectra of pyrene with PEG2K-PHis sequences were overlapped, but the decrease in the emission peak at 505 nm with the decrease of the copolymer concentration due to the excimer formation could be observed.

The intensity ratio between I_excimer_ (peak intensity at 505 nm characteristic of the formed excimers) and I_3_ (peak intensity at 386 nm characteristic of the pyrene monomers) decreased with the decrease in copolymer concentration, reaching a constant variation in each studied case, showing the concentration values where the copolymers no longer had the ability to form micelles.

**Figure 1:**
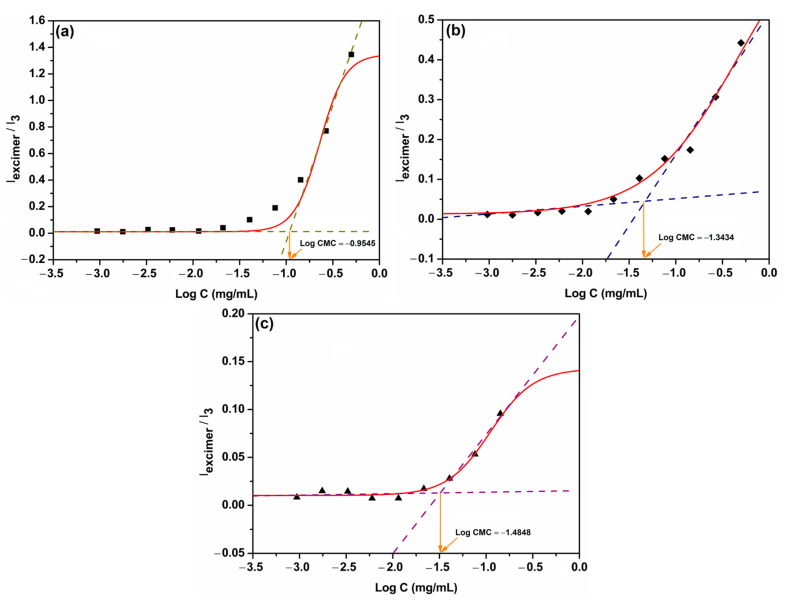
The ratio of the intensity (I_excimer_/I_3_) of the pyrene to the logarithm of the concentration of the copolymers PEG2K-PHis20 (**a**), PEG2K-PHis26 (**b**), and PEG2K-PHis32 (**c**).

Fitting the emission intensities with the concentration profiles, the determined CMC values (Figure 1) for the investigated PEG2K-PHis sequences corresponded to 0.111 mg/mL for PEG2K-PHis20, 0.045 mg/mL for PEG2K-PHis26, and 0.032 mg/mL for PEG2K-PHis32.

The decrease in CMC values from 0.111 mg/mL to 0.032 mg/mL with the increase in the PHis moiety length of the investigated copolymers was due to the general tendency for the hydrophobic part of these systems to strongly govern the assembly into micelles [34]. The obtained CMC values for the studied SPPS PEG2K-PHis sequences were comparable with the previously reported results on similar systems obtained via polymerization to PHis and subsequent coupling of the PEG2K unit. The obtained CMC values for the studied SPPS-synthesized PEG2K-PHis sequences were in agreement with or slightly higher than the previously reported CMC results on similar systems obtained via polymerization of PHis and subsequent coupling of the PEG2K unit [34] in the studied pH range (pH 7.4). The small differences could be explained by the presence of two additional amino acids (cysteine as a linker between PHis and PEG, as well as terminal lysine) in the SPPS sequences, which may induce the instabilities of the micelles at the investigated pH value.

### 3.3. Dynamic Light Scattering (DLS) and Zeta Potential (ζ)

**Figure 2:**
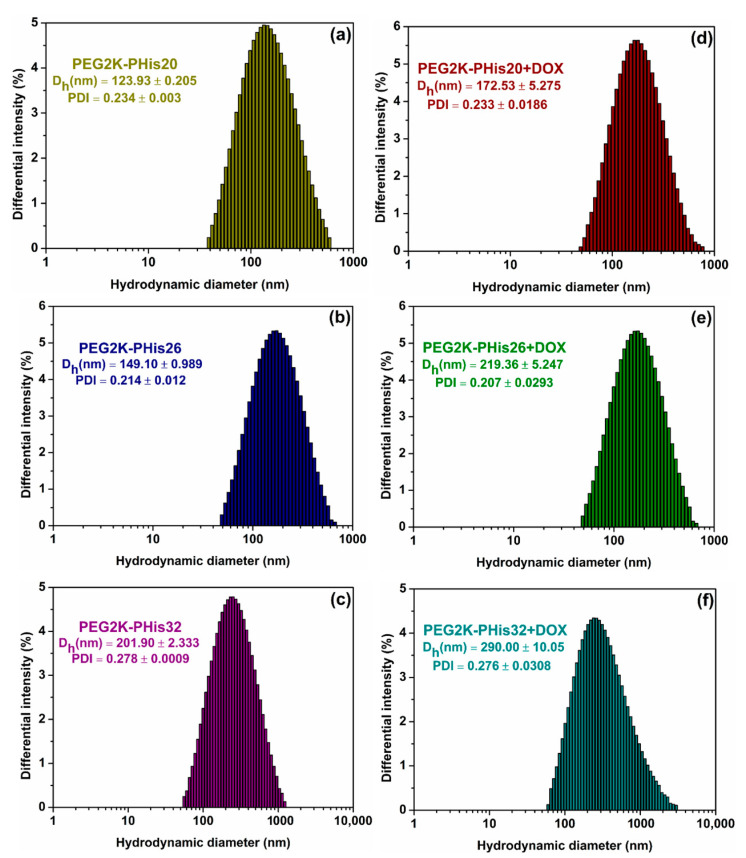
Distributions of average hydrodynamic diameters measured using dynamic light scattering (DLS) of unloaded micelles (**a**–**c**) and those loaded with DOX (**d**–**f**).

The structural changes and mean hydrodynamic diameters of the unloaded and DOX-loaded micelles above the CMC values were monitored using DLS (Figure 2, Table S1). In the case of unloaded micelles (Figure 2a–c, Table S1), the DLS histograms revealed uniform assemblies with a clear increasing tendency regarding the hydrodynamic diameter with the increase in the length of hydrophobic polyhistidine moiety in the copolymer structure. The PEG2K-PHis20 sample displayed a hydrodynamic diameter of 123.93 ± 0.205 nm with a polydispersity of 0.234 ± 0.003 nm, followed by the PEG2K-PHis26 sample with a hydrodynamic diameter of 149.10 ± 0.989 nm (polydispersity of 0.214 ± 0.012), and reaching a hydrodynamic diameter of 202 ± 2 nm for the PEG2K-PHis32 sample (polydispersity of 0.278 ± 0.001).

Due to the fact that the hydrophobic polyhistidine moieties are capable of accommodating drugs during the micellization process, we next investigated the DOX-loaded micelles and compared them with the previous experiment. Loading the maximum amount of DOX into the micelles considerably altered the resulted hydrodynamic diameters (Figure 2d–f). A significant characteristic increase [53,54] in the hydrodynamic diameters for all the investigated samples in comparison to the free micelles was observed. By analyzing the histograms in Figure 2c–e and Table S1, it was observed that in the case of the PEG2K-PHis20 + DOX sample, the average determined hydrodynamic diameter was 172 ± 5 nm with a polydispersity of 0.23 ± 0.02, the PEG2K-PHis26 + DOX sample presented a hydrodynamic diameter of 219 ± 5 nm (polydispersity of 0.21 ± 0.03), while the PEG2K-PHis32 + DOX sample reached a hydrodynamic diameter of 290 ± 10 nm (polydispersity of 0.28 ± 0.03).

The observed increase in hydrodynamic diameters revealed the successful loading of a biologically relevant drug, while the results were comparable with the previously reported DOX-loaded polyhistidine-based systems [40].

**Figure 3:**
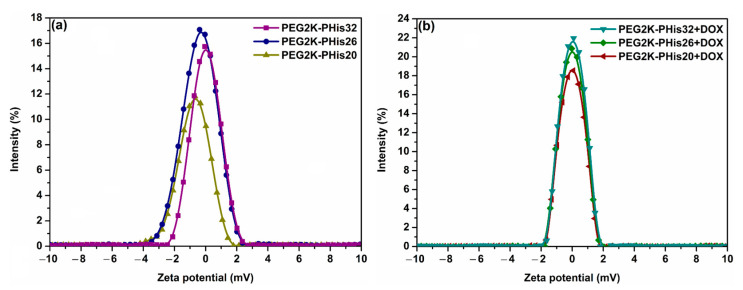
Mean zeta potential distribution curves of the unloaded micelles (**a**) and DOX-loaded micelles (**b**).

The mean measured values of zeta potential for the unloaded and DOX-loaded micelles are represented in Figure 3 and Table S1. Since the PEG micelle component has a negative zeta potential [55] and polyhistidine moiety has a positive potential at a pH value of 7.4, the ratio of the length of these two entities in the copolymer structure strongly influences the overall zeta potential value of the formed micelles. In the case of the unloaded micelles, the increase in the length of the polyhistidine unit from 20 to 32 amino acids in the copolymer structure led to a linear variation of the zeta potential from −9 ± 3 mV to 0.47 ± 0.30 mV (Figure 3a). Loading micelles with positively charged DOX molecules [54] led to an increase in zeta potential for all the investigated samples (Figure 3b, Table S1).

Interestingly, a major difference between the unloaded (−9 ± 3 mV) and DOX-loaded (0.23 ± 0.07 mV) zeta potential values was observed in the case of the PEG2K-PHis20 micelles. This could be explained by the ratio between the length of the polyhistidine unit and the amount of loaded DOX molecules within the micelles reported earlier [54].

### 3.4. Scanning Transmission Electron Microscopy (STEM) of Micelles

**Figure 4:**
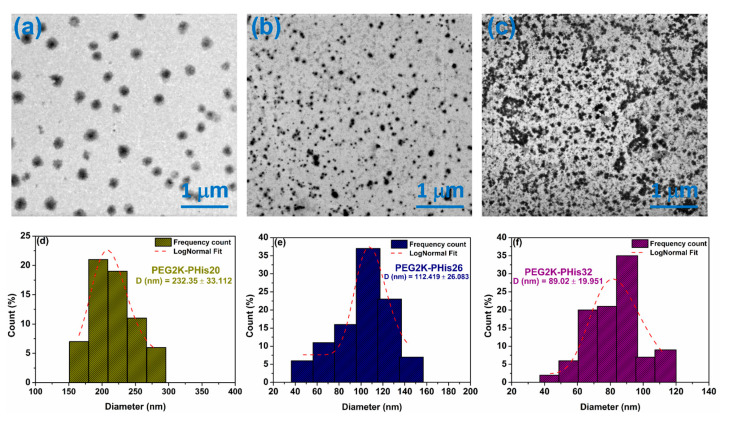
Scanning transmission electron microscopy images of unloaded micelles (**a**–**c**) and distribution of diameters (**d**–**f**).

Scanning transmission electron microscopy was used to highlight the morphology of the formed micelles. Figure 4 shows examples of STEM images of the PEG2K-PHis sequences, revealing their assembly into spherical nano-sized particles with a calculated diameter distribution for each investigated sample. In the case of the PEG2K-PHis20 sample (Figure 4a), a core–shell structure could be observed, with an average diameter of 232 ± 33 nm, along with a strong contrast of the hydrophobic side of the micelles due to the polyhistidine core, which has a higher electron density in comparison with the hydrophilic PEG shell.

The PEG2K-PHis26 sample displayed an average diameter of 112 ± 26 nm (Figure 4e), while the PEG2K-PHis32 sample had an average diameter of 89 ± 20 nm (Figure 4f). By analyzing the diameters of the unloaded micelles, we observed that the average diameter decreased with the length of the PHis moiety, which contradicted the DLS data. This fact might have been due to the higher stability of the micelles with longer PHis owing to the stronger hydrophobic interactions involved in the micelle core assembly. The more compact core assembly was also reflected in the higher contrast of the core in the STEM images of the PEG2K-PHis26 and PEG2K-PHis32 samples (Figure 4b,c).

The lower stability of the PEG2K-PHis20 micelles could generate bigger aggregates during the drying process on carbon-coated copper meshes for the STEM sample preparation. This drying protocol could also explain the overall smaller micelle diameters determined in STEM in comparison to DLS, where the samples were in the hydrated state in solutions.

**Figure 5:**
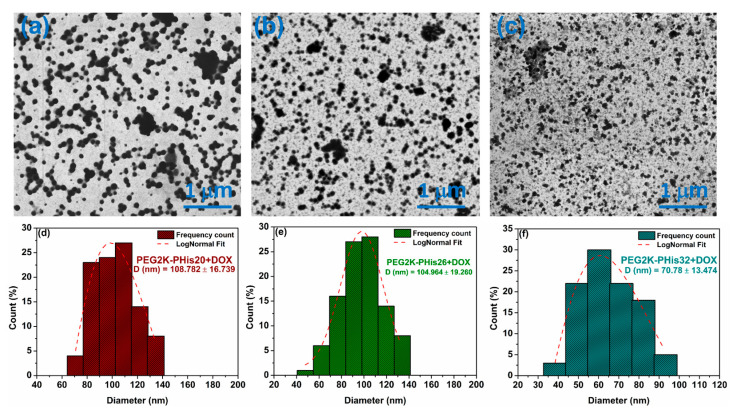
Scanning transmission electron microscopy images of DOX-loaded micelles (**a**–**c**) and distribution of diameters (**d**–**f**).

In the case of DOX-loaded micelles, the STEM images displayed uniform spherical assemblies with much higher contrast due to the successful DOX incorporation (Figure 5). Interestingly, all samples in STEM images (Figure 5a–c) revealed a gradual decrease in average diameters compared with the unloaded micelles (Figure 5d–f, PEG2K-PHis20 + DOX with an average diameter of 109 ± 17 nm, PEG2K-PHis26 + DOX with an average diameter of 105 ± 19 nm, and PEG2K-PHis32 + DOX with an average diameter of 71 ± 13 nm), indicating strong unspecific hydrophobic interactions of PHis with the DOX molecules [56]. Additionally, DOX may form compact nanocrystals inside the PHis moiety, where this phenomenon is observed with drugs in a similar environment [57], all leading to compaction of the inner core.

The compaction of the micelles by DOX molecules also induced unexpected enhanced stability of the assemblies in all three studied samples. Since the DOX loading considerably shifted the zeta potential values of the micelles to almost neutral, it could facilitate easier aggregation to larger particles due to low electrostatic repulsion [58]; this process was not observed during the performed STEM experiments.

### 3.5. Encapsulation Efficiency (EE) and Drug Loading (DL) of DOX in Micelles

**Figure 6:**
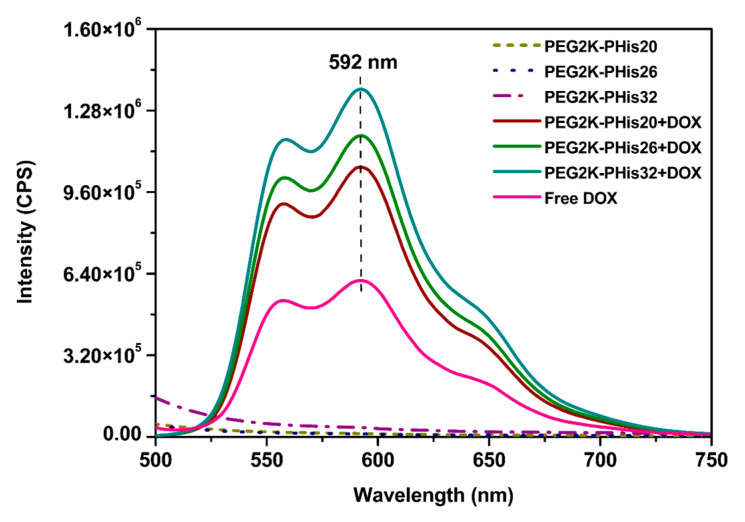
Overlapping the emission spectra of the unloaded micelles and DOX-loaded micelles of the free DOX with the highlighting of the emission peak given by DOX at 592 nm.

Investigations of the amount of DOX in copolymer micelles were performed in 1X PBS at pH 7.4 by monitoring the characteristic DOX emission maximum at 592 nm (Figure 6). Unloaded micelles did not show any emission, while purified DOX-loaded micelles displayed the emission peak characteristic of DOX with the intensities being strongly dependent on the amount of loaded DOX for each sample.

To quantitatively evaluate the amount of micelle-loaded DOX, the signal intensity at 592 nm and the calibration curve of free DOX in 1X PBS at pH 7.4 were utilized (Figure S5). The determined concentrations were used to calculate the encapsulation efficiency (EE) and drug loading (DL) of the drug for each investigated sample (Table 1).

The amount of DOX incorporated into micelles gradually increased with the growth in length of the polyhistidine site of the copolymer, which was strongly driven by the block copolymer structure. Higher amounts of loaded DOX in micelles with a longer PHis site correlated with the STEM data on the higher degree of DOX molecule compaction in the corresponding micelles.

### 3.6. pH-Triggered Doxorubicin Release Study

**Figure 7:**
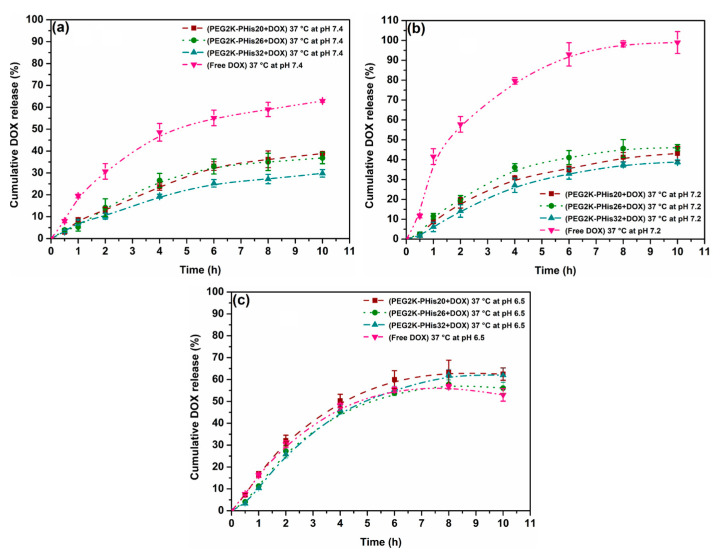
Drug release profiles of DOX-loaded micelles and free DOX in 1X PBS at pH 7.4 (**a**), pH 7.2 (**b**), and pH 6.5 (**c**) at 37 °C.

A DOX release study from three types of loaded copolymer micelles at 37 °C that involved triggering a change in pH by simulating the extracellular environment of tumor cells with a pH range of 6.5 to 6.9 [59] was performed. In a previous report, Bae and others reported that in the case of PHis-based copolymeric assemblies, a pH variation that protonates or deprotonates the two nitrogen atoms in the imidazole rings of polyhistidine leads to either the assembly of copolymers into micelles or their disassembly [32,34,35]. The investigated pH values in the study were 7.4 (the value at which DOX-loaded micelles were assembled), 7.2, and 6.5 in correspondingly adjusted 1X PBS buffers. Importantly, the release of DOX at pH = 7.0 was omitted due to the fact that DOX molecules underwent a dimerization process at this pH value, leading to DOX precipitation [60]. Additionally, the release time of the study was limited to 24 h to avoid the prolonged exposure of DOX in an aqueous solution, which also may lead to partial precipitation and inaccurate results. The amount of released DOX was determined indirectly via fluorescence measurements of the corresponding solutions (Table S2) using a standard calibration curve of DOX in 1X PBS buffer for each pH value (Figure S6). Figure 7 shows the DOX release profiles of the three types of loaded micelles at three pH values having free DOX at a temperature of 37 °C as a control in each case.

It could be observed that after 10 h, the cumulative DOX release from PEG-PHis32-based nanoparticles was roughly 20–24% at pH 7.4 and slightly higher at pH 7.2, and was always smaller than for the PEG2K-PHis20 + DOX, PEG2K-PHis26 + DOX, and free DOX samples. These findings revealed that PEG-PHis diblock copolymers loaded with DOX are sensitive to tiny pH variations toward acidic pH on one hand, and on the other hand, once the PHis length increased, the loaded system with DOX became more stable at a physiological pH of 7.4. At pH 6.5, the cumulative release of PEG2K-PHis20 + DOX and PEG2K-PHis26 + DOX samples was equivalent to those of the PEG2K-PHis32 + DOX sample, but was at least two times higher than at pH 7.2 and pH 7.4, where the start of a release plateau was observed at 6 h for all samples. At pH 7.4, the obtained DOX release was triggered mainly by the temperature value, but also by the variation in the PHis side length due to only partial protonation of polyhistidine, leading to a low percentage release [34]. A small increase in DOX release was observed at pH 7.2 for all the investigated samples (Table S2) due to a higher percentage of polyhistidine protonation. When the pH dropped to 6.5, the polyhistidine moiety in all the samples was completely protonated; therefore, the DOX release rate significantly increased compared with the values at pH 7.2 (Figure 7c, Table S2). This latter aspect is of particular interest since, at pH 6.5, which is the lower limit of extracellular pH in the case of tumor cells [59], the micelles released over 50% of DOX, thus demonstrating the potential of the investigated systems as efficient platforms for the release of drugs in an extracellular tumor environment.

### 3.7. In Vitro Cell Studies

**Figure 8:**
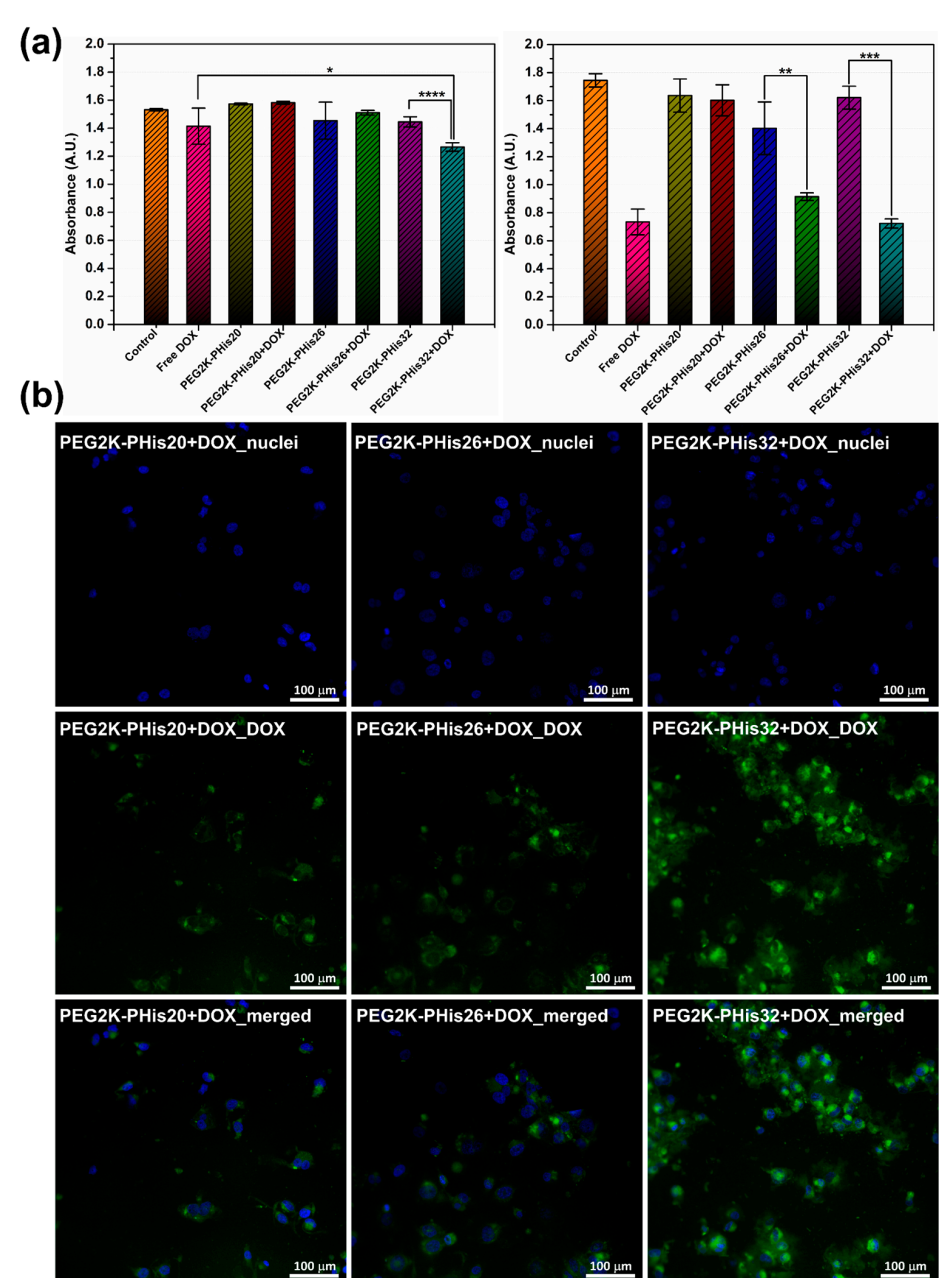
The effects of DOX, unloaded micelles, and DOX-loaded micelles on MDA-MB-231 cells. (**a**) Cellular viability at 24 h (left) and 48 h (right) expressed as absorbance; (**b**) representative immunofluorescence images at 48 h for the three types of DOX loaded micelles; images with the highlighting of cell nuclei (blue), images with the highlighting of DOX auto-fluorescence, and the merging of the two images for each sample. * *p* < 0.05, ** *p* < 0.005, *** *p* < 0.0005, **** *p* < 0.00005. Pictures were acquired at 20× magnification.

To further evaluate the stability, cytotoxicity, and drug release properties of PEG-PHis micelles, we performed an in vitro assessment of the prepared systems on human triple-negative cells of the MDA-MB-231 cell line (Figure 8).

**Figure 9:**
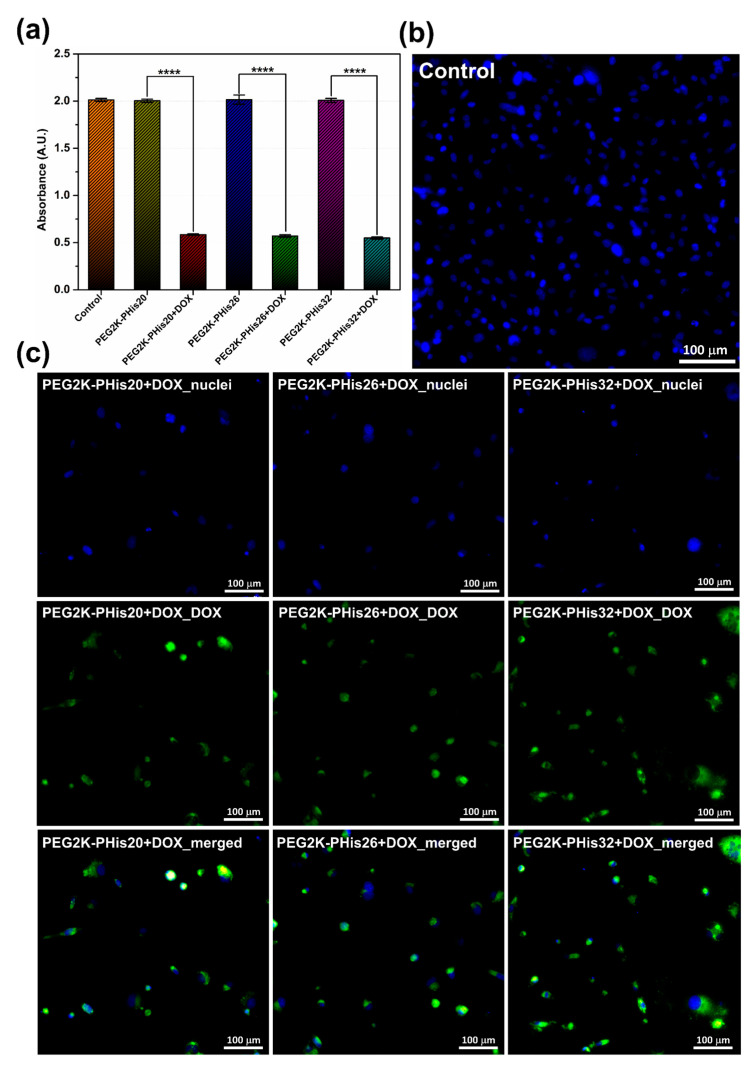
Cell viability and immunofluorescence at 72 h: (**a**) MDA-MB-231 viability at 72 h expressed as absorbance for the control, unloaded micelles, and DOX-loaded micelles; (**b**) immunofluorescence image for the nuclei of MBA-MB-231 cells line used as a control; (**c**) immunofluorescence images for the three types of micelles loaded with DOX; images with the highlighting of cell nuclei, images with the highlighting of DOX-loaded micelles, and the merging of the two images for each sample. **** *p* < 0.0001. Pictures were acquired at 20× magnification.

At 24 h after the treatment administration of MDA-MB-231 cells (pH = 7.40), the cell viability was measured by quantifying the absorbance in a multi-plate reader; DOX loaded in the PEG2K-PHis32 + DOX sample significantly reduced the cell viability relative to the unloaded PEG2K-PHis32 or free DOX (Figure 8a (left)). At 48 h after the treatment induction (Figure 8a (right)), the cell viability was significantly impaired in free DOX, PEG2K-PHis26 + DOX, and PEG2K-PHis32 + DOX, but remained unaffected by the PEG2K-PHis20 + DOX treatment. However, the impact of PEG2K-PHis26 + DOX had a lower magnitude relative to free DOX or PEG2K-PHis32 + DOX. The presence of unloaded micelles did not significantly reduce cell viability. Immunofluorescence investigations at 48 h from treatment administration showed the increased emission of DOX loaded in PEG2K-PHis32 + DOX relative to PEG2K-PHis26 + DOX or PEG2K-PHis20 + DOX (Figure 8b). Unloaded micelles did not present immunofluorescence signals (Figure S7). After 72 h of incubation (pH = 7.20) with the unloaded micelles, the cell viability had not been modulated by the presence of unloaded micelles relative to the untreated control group (Figure 9a).

In contrast, the cell viability was significantly impaired in the presence of all the investigated DOX-loaded micelles (Figure 9a). At the investigated concentration of DOX-loaded micelles, the cell viability decreased similarly, showing the same low levels of viability, suggesting that under these experimental conditions, the loaded DOX was released from all tested micelles (Figure 9a). The observed fluorescence signal of DOX supported the viability data, as the intensity of the fluorescence signal was at similar levels across the treated groups. Immunofluorescent data confirm the lack of auto-fluorescence in the control group (Figure 9b) and the presence of DOX in the loaded micelles treated groups (Figure 9c).

**Figure 10:**
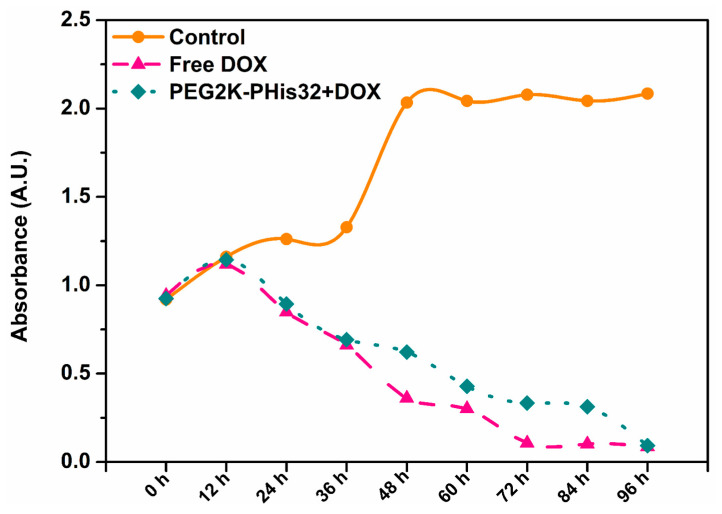
The dynamics of cell viability. Each measurement was done in 8 wells per group and per time point.

Our results indicated a higher zeta potential of PEG2K-PHis32 + DOX, which induced higher stability compared with the other two loaded micelles, and the fact that PEG2K-PHis32 + DOX presented the smallest average diameter (Figure 5); these features provide an advantage in tissue penetration. In the next experimental setup, we tested the dynamics of the cell population under free DOX or PEG2K-PHis32 + DOX treatments. The dynamics of the cell population provided information regarding the effects of released DOX from PEG2K-PHis32 + DOX relative to free DOX. Cell viability investigations every 12 h revealed the dynamics of the cell population, as measured previously using absorbance in a multi-plate reader (Figure 10).

While the cell viability (which reflects cell numbers) increased over time until it reached a plateau in the control (untreated) group, in the DOX-treated groups, the cell viability decreased and, at 96 h after the treatment administration, there was no difference in the cell viability of free DOX relative to PEG2K-PHis32 + DOX. Moreover, at 72 h, the cell viability reached a plateau in the free-DOX-treated group, as it presented similar values between 72 h and 96 h.

## 4. Conclusions

Three designed polyhistidine-PEG diblock copolymers prepared using SPPS were investigated for the formation of micellar structures, the ability to incorporate controlled amounts of DOX, and the pH-dependent drug release studies. For all the examined samples, the critical micelle concentrations were determined using pyrene fluorescence as the standard compound and the values were compared with similar copolymeric systems prepared using polyhistidine polymerization. The obtained micelles were morphologically analyzed using DLS, zeta potential measurements, and scanning transmission electron microscopy, revealing uniform assemblies with sizes being dependent on the length of the polyhistidine side. The encapsulation efficiency of DOX-loaded micelles and the drug-loading values were determined, together with the controlled pH-dependent DOX release studies, highlighting the fact that the investigated micelles released an amount of DOX of over 50% in the pH range of the malignant extracellular environment. This behavior, together with the outstanding cytotoxicity of these types of micelles determined on the MDA-MB-231 breast tumor cell line, was in agreement with earlier reported polyhistidine-PEG systems, thus making the investigated SPPS synthesized sequences suitable systems for the transport and release of biologically active molecules in the malignant extracellular environment.

## Figures and Tables

**Table 1:** Encapsulation efficiency (EE) and drug loading (DL) of DOX in micelles.

Sample Name	EE (%)	DL (%)
PEG2K-PHis20 + DOX	52.98	8.11
PEG2K-PHis26 + DOX	60.51	9.16
PEG2K-PHis32 + DOX	71.31	10.62

## Data Availability

The data presented in this study are available upon reasonable request from the corresponding author.

## Supplementary Materials

The following supporting information can be downloaded at: https://www.mdpi.com/article/10.3390/nano12111798/s1, Figure S1. The mass spectrum (a) and HPLC chromatogram (b) of PEG2K-PHis20 copolymer; Figure S2. The HPLC chromatogram (a) and mass spectrum (b) of PEG2K-PHis26 copolymer; Figure S3. The mass spectrum (a) and HPLC chromatogram (b) of PEG2K-PHis32 copolymer; Figure S4. The potentiometric titration curves of the three copolymers and determination of their pK_a_: PEG2K-PHis20 (a), PEG2K-PHis26 (b) and PEG2K-PHis32 (c); Figure S5. Pyrene emission spectra with different copolymer concentrations, PEG2K-PHis20 (a), PEG2K-PHis26 (b) and PEG2K-PHis32 (c); Figure S6. Free DOX calibration curve in 1X PBS at pH 7.4 (a,b), pH 7.2 (c,d) respectively pH 6.5 (e) and (f) by fluorescence with DOX emission peak at 592 nm; Figure S7. Representative immunofluorescence images for the three types of unloaded micelles, where it can be seen that these micelles do not show fluorescence, and fluorescence is given only by the nuclei of cells stained with NucBlue (Invitrogen); Table S1. Dynamic light scattering (DLS) and zeta potential of unloaded micelles (PEG2K-PHis20, PEG2K-PHis26 and PEG2K-PHis32) and those loaded with doxorubicin (PEG2K-PHis20 + DOX, PEG2K-PHis26 + DOX and PEG2K-Phis32 + DOX) in 1X PBS at pH 7.4; Table S2. The average percentages of DOX release from micelles concomitantly with Free DOX at different times and three pH values of 1X PBS at a temperature of 37 °C. Release studies were performed in triplicate.
