# Supplementary material for: Solid-Phase Synthesized Copolymers for the Assembly of pH-Sensitive Micelles Suitable for Drug Delivery Applications
Source: Nanomaterials (Basel). 2022 May 24;12(11):1798. doi: 10.3390/nano12111798 (PMC9181997; doi:10.3390/nano12111798)
Supplement: Supplementary file 1 [file nanomaterials-12-01798-s001.zip › nanomaterials-1700139 - Supplementary Materials-v1.pdf]

nanomaterials

Supporting Information

Solid-Phase Synthesized Copolymers for the Assembly of pH-Sensitive Micelles Suitable for Drug Delivery Applications

Razvan Ghiarasim [1], Crina Elena Tiron [2], Adrian Tiron [2], Mihail-Gabriel Dimofte [2], Mariana Pinteala [1,]* and Alexandru Rotaru [1,]*

[1] Centre of Advanced Research in Bionanoconjugates and Biopolymers, "Petru Poni" Institute of Macromolecular Chemistry, 41A Grigore Ghica Voda Alley, 700487 Iasi, Romania; ghiarasim.razvan@icmpp.ro

[2] TRANSCEND Centre, Regional Institute of Oncology, 2–4 General Henri Mathias Berthelot Street, 700483 Iasi, Romania; transcendctiron@iroiasi.ro (C.E.T.); adrian.tiron@iroiasi.ro (A.T.); mihail.dimofte@umfiasi.ro (M.-G.D.)

* Correspondence: pinteala@icmpp.ro (M.P.); rotaru.alexandru@icmpp.ro (A.R.)

Content

S1.	Physico-Chemical Characterization of the Three Copolymers By High-Performance Liquid Chromatography (HPLC) And Mass Spectroscopy (MS)	Figures S1–S3
S2.	The pK$_a$'s of the Three Copolymers	Figure S4
S3.	Critical Micelle Concentration (CMC)	Figure S5
S4.	Dynamic Light Scattering (DLS) and Zeta Potential	Table S1
S5.	Doxorubicin Calibration Curves at Different Values of pH by Fluorescence and the Percentages of DOX Released at Different pH Values	Figure S6<br>Table S2
S6.	Representative Immunofluorescence Images for the Three Types of Unloaded Micelles	Figure S7

S1. Physico-Chemical Characterization of the Three Copolymers by High-Performance Liquid Chromatography (HPLC) And Mass Spectroscopy (MS)

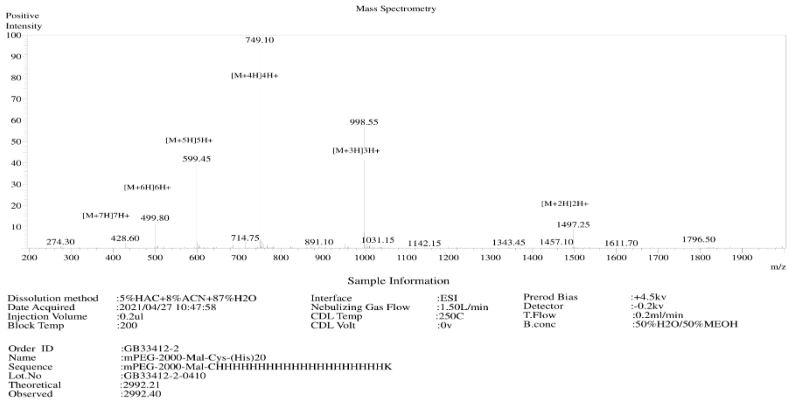

(a)

Figure S1. *Cont.*

Sample Information

Order ID :GB33412-2
Name :mPEG-2000-Mal-Cys-(His)20
Sequence :mPEG-2000-Mal-CHHHHHHHHHHHHHHHHHHHHK
Lot. No :GB33412-2-0410
Pump A :0.1%Trifluoroacetic in 100% water
Pump B :0.1%Trifluoroacetic in 100% acetonitrile
Total Flow :1ml/min
Wavelength :214nm
Analytial column type :SHIMADZU Inertsil ODS-SP(4.6*250mm*5um)
Dissolution method :100%H2O
Inj. Volume : 25ul

Time	Module	Action	Value
0.01	Pumps	B.Conc	10
20.00	Pumps	B.Conc	30
33.00	Pumps	B.Conc	100
38.00	Pumps	B.Conc	100
40.00	Pumps	B.Conc	10
50.00	Controller	Stop	

Chromatogram

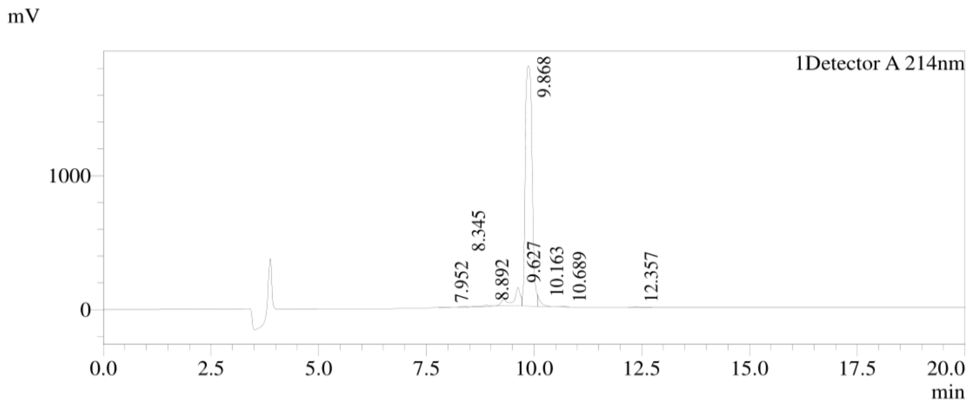

Peak Table

Detector A 214nm

Peak#	Ret. Time	Area	Height	Area%
1	7.952	10510	1095	0.048
2	8.345	20751	2896	0.095
3	8.892	76179	9034	0.348
4	9.627	1673685	139738	7.653
5	9.868	19684728	1796441	90.012
6	10.163	374639	28915	1.713
7	10.689	4679	785	0.021
8	12.357	23916	2029	0.109
Total		21869086	1980934	100.000

(**b**)

Figure S1. The mass spectrum (**a**) and HPLC chromatogram (**b**) of PEG2K-PHis20 copolymer.

Sample Information

Order ID	:GB33412-3
Name	:mPEG-2000-Mal-Cys-(His)26
Sequence	:mPEG-2000-Mal-CHHHHHHHHHHHHHHHHHHHHHHHHHHK
Lot. No	:GB33412-3-0410
Pump A	:0.1%Trifluoroacetic in 100% water
Pump B	:0.1%Trifluoroacetic in 100% acetonitrile
Total Flow	:1ml/min
Wavelength	:214nm
Analytial column type	:SHIMADZU Inertsil ODS-SP(4.6*250mm*5um)
Dissolution method	:100%H2O
Inj. Volume	: 10ul

Time	Module	Action	Value
0.01	Pumps	B.Conc	5
25.00	Pumps	B.Conc	30
33.00	Pumps	B.Conc	100
38.00	Pumps	B.Conc	100
40.00	Pumps	B.Conc	5
50.00	Controller	Stop	

Chromatogram

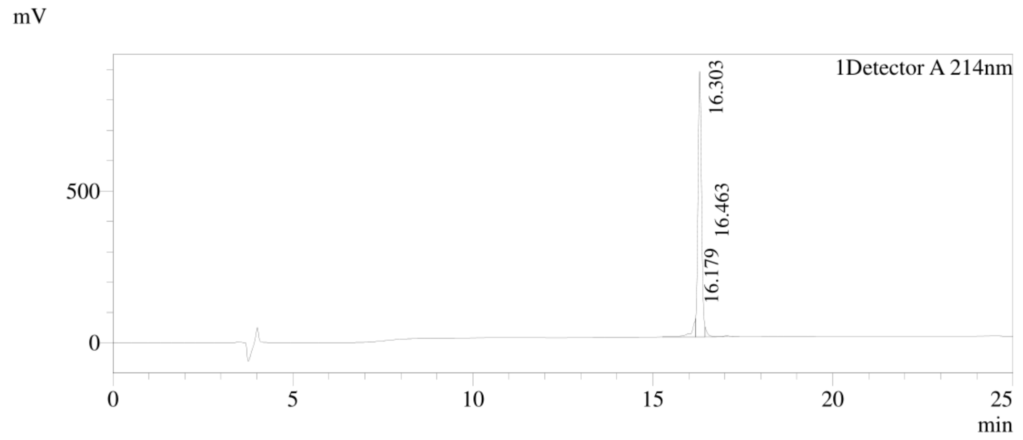

Peak Table

Detector A 214nm

Peak#	Ret. Time	Area	Height	Area%
1	16.179	434797	51192	6.628
2	16.303	5928019	875575	90.369
3	16.463	196964	29100	3.003
Total		6559780	955867	100.000

(**a**)

Figure S2. *Cont.*

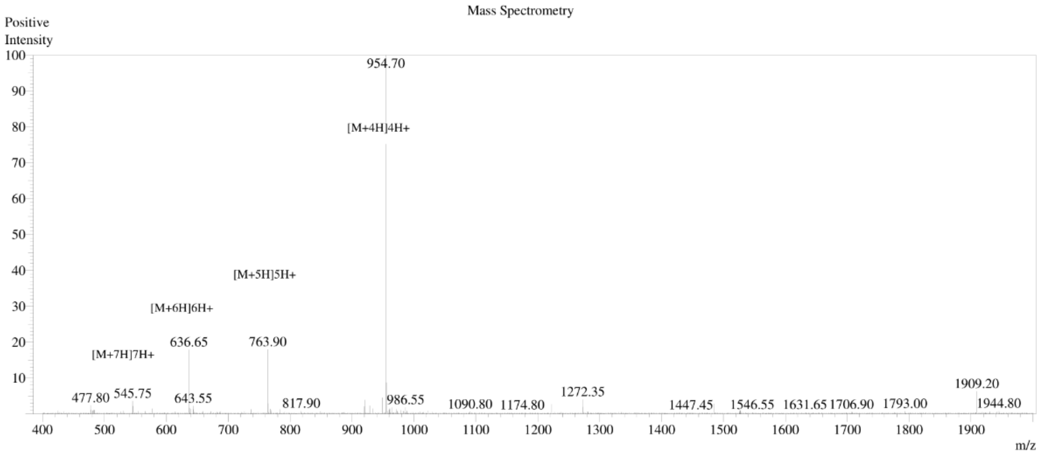

Sample Information

Dissolution method	:5%HAC+8%ACN+87%H2O	Interface	:ESI	Prerod Bias	:+4.5kv
Date Acquired	:2021/04/29 16:55:52	Nebulizing Gas Flow	:1.50L/min	Detector	:-0.2kv
Injection Volume	:0.2ul	CDL Temp	:250C	T.Flow	:0.2ml/min
Block Temp	:200	CDL Volt	:0v	B.conc	:50%H2O/50%MEOH

Order ID	:GB33412-3
Name	:mPEG-2000-Mal-Cys-(His)26
Sequence	:mPEG-2000-Mal-CHHHHHHHHHHHHHHHHHHHHHHHHHHHK
Lot.No	:GB33412-3-0410
Theoretical	:3815.07
Observed	:3814.80

(**b**)

Figure S2. The HPLC chromatogram (**a**) and mass spectrum (**b**) of PEG2K-PHis26 copolymer.

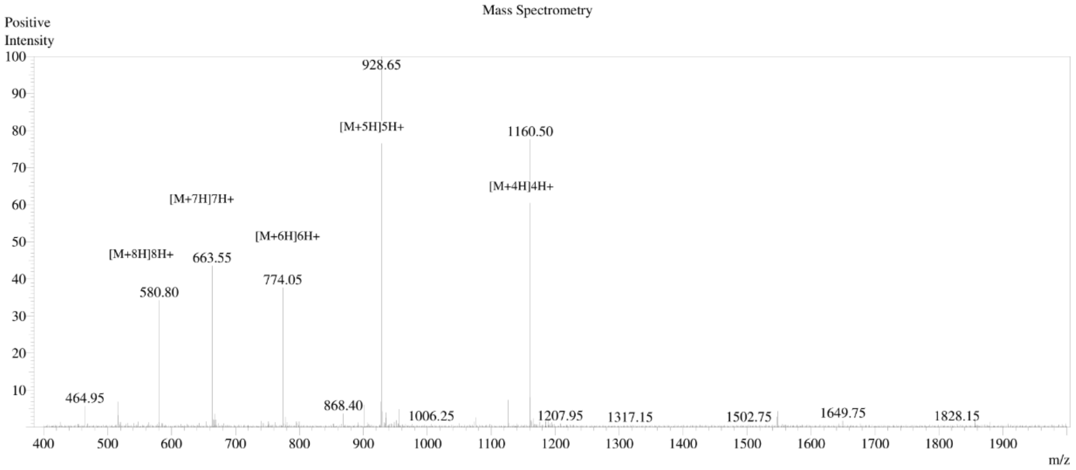

Sample Information

Dissolution method	:5%HAC+8%ACN+87%H2O	Interface	:ESI	Prerod Bias	:+4.5kv
Date Acquired	:2021/04/29 17:17:35	Nebulizing Gas Flow	:1.50L/min	Detector	:-0.2kv
Injection Volume	:0.2ul	CDL Temp	:250C	T.Flow	:0.2ml/min
Block Temp	:200	CDL Volt	:0v	B.conc	:50%H2O/50%MEOH

Order ID	:GB33412-4
Name	:mPEG-2000-Mal-Cys-(His)32
Sequence	:mPEG-2000-Mal-CHHHHHHHHHHHHHHHHHHHHHHHHHHHHHHHHHK
Lot.No	:GB33412-4-0410
Theoretical	:4637.94
Observed	:4638.25

(a)

Figure S3. *Cont.*

Sample Information

Order ID :GB33412-4
Name :mPEG-2000-Mal-Cys-(His)32
Sequence :mPEG-2000-Mal-CHHHHHHHHHHHHHHHHHHHHH HHHHHHHHHHHK
Lot. No :GB33412-4-0410
Pump A :0.1%Trifluoroacetic in 100% water
Pump B :0.1%Trifluoroacetic in 100% acetonitrile
Total Flow :1ml/min
Wavelength :214nm
Analytial column type :SHIMADZU Inertsil ODS-SP(4.6*250mm*5um)
Dissolution method :100%H2O
Inj. Volume : 10ul

Time	Module	Action	Value
0.01	Pumps	B.Conc	5
25.00	Pumps	B.Conc	30
33.00	Pumps	B.Conc	100
38.00	Pumps	B.Conc	100
40.00	Pumps	B.Conc	5
50.00	Controller	Stop	

Chromatogram

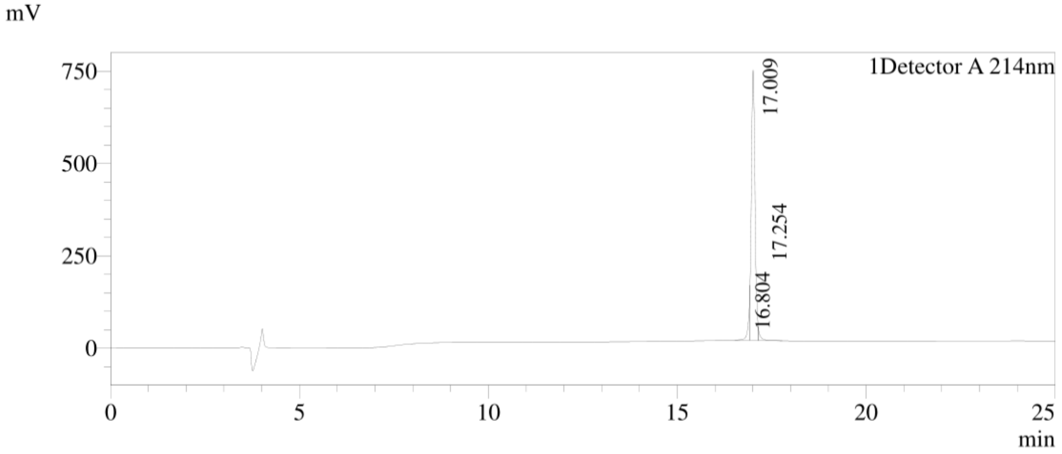

Peak Table

Detector A 214nm

Peak#	Ret. Time	Area	Height	Area%
1	16.804	409546	7007	7.556
2	17.009	4894886	732003	90.304
3	17.254	116045	5327	2.141
Total		5420476	744337	100.000

(b)

Figure S3. The mass spectrum (**a**) and HPLC chromatogram (**b**) of PEG2K-PHis32 copolymer.

S2. The pK$_a$'s of the Three Copolymers

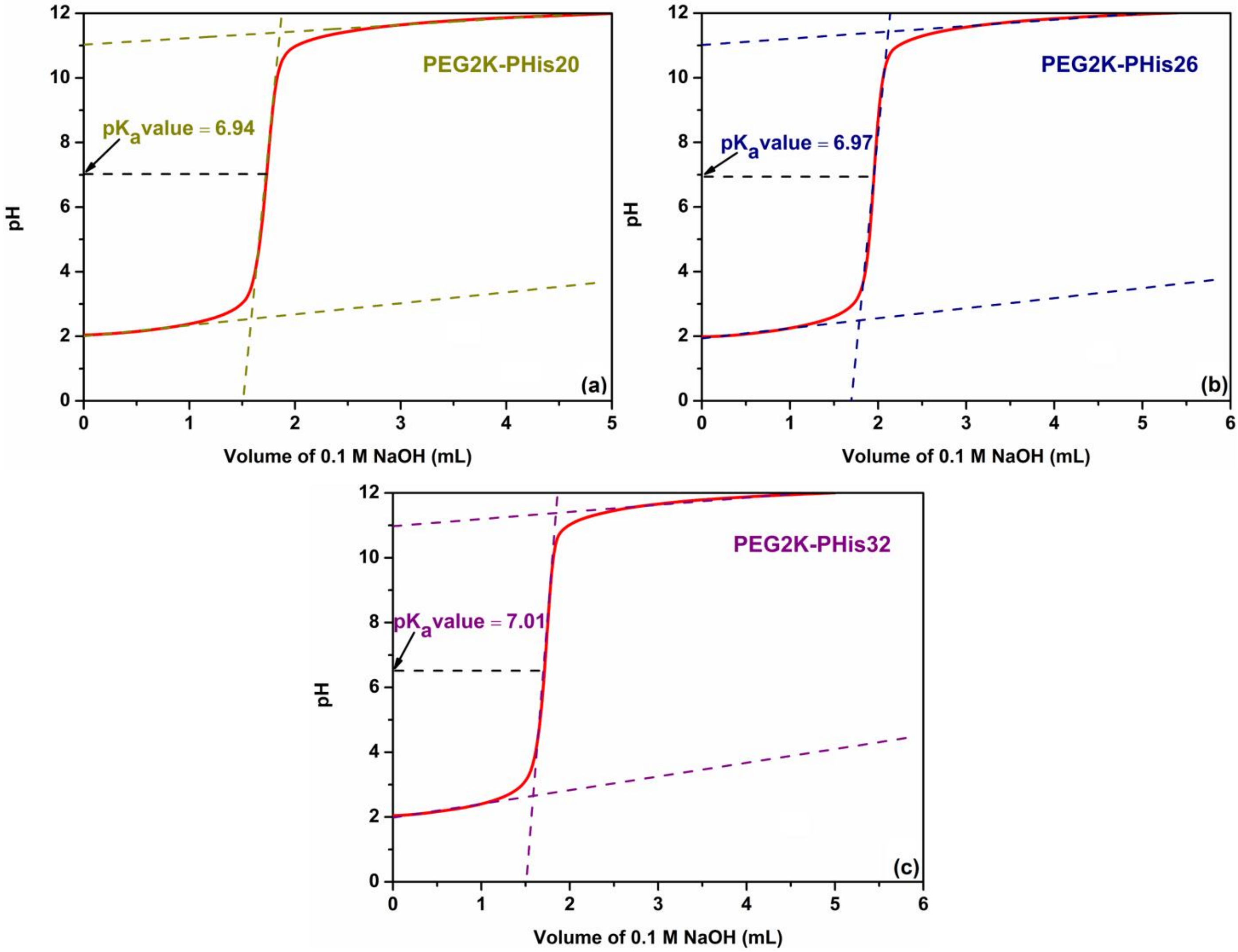

Figure S4. The potentiometric titration curves of the three copolymers and determination of their pK$_a$: PEG2K-PHis20 (**a**), PEG2K-PHis26 (**b**) and PEG2K-PHis32 (**c**).

S3. Critical Micelle Concentration (CMC)

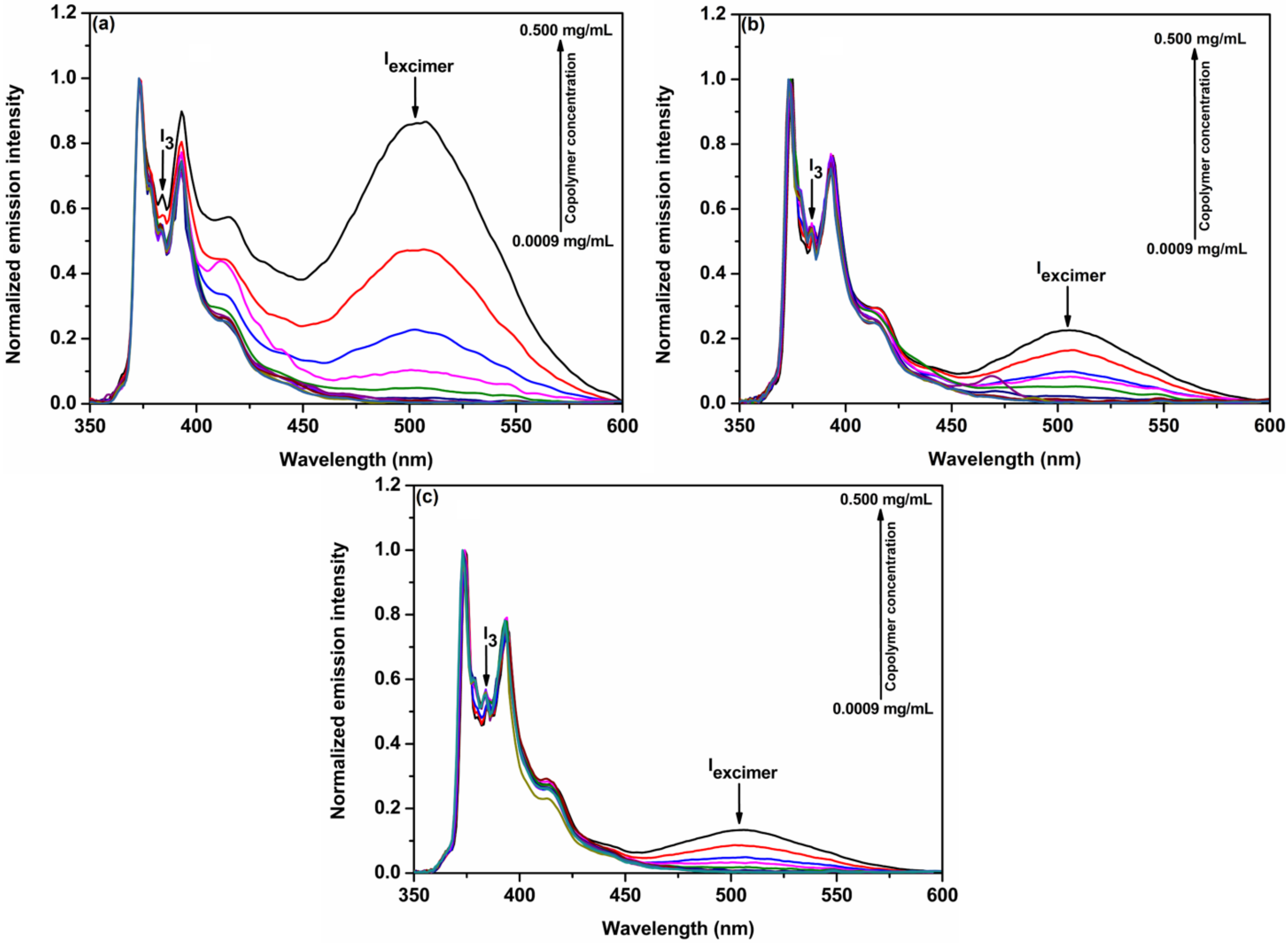

Figure S5. Pyrene emission spectra with different copolymer concentrations, PEG2K-PHis20 (**a**), PEG2K-PHis26 (**b**) and PEG2K-PHis32 (**c**).

S4. Dynamic Light Scattering (DLS) and Zeta Potential

Table S1. Dynamic light scattering (DLS) and zeta potential of unloaded micelles (PEG2K-PHis20, PEG2K-PHis26 and PEG2K-PHis32) and those loaded with doxorubicin (PEG2K-PHis20 + DOX, PEG2K-PHis26 + DOX and PEG2K-Phis32 + DOX) in 1X PBS at pH 7.4.

Sample Name	D_h by DLS * (nm)	Average D_h (nm ± SD *)	PDI *	Average PDI * ± SD *	ζ Potential * (mV)	Average ζ Potential * ± SD *
PEG2K-PHis20	124.2	123.93 ± 0.205	0.236	0.234 ± 0.003	−4.74	−8.97 ± 2.992
	123.7		0.238		−11.21	
	123.9		0.229		−8.97	
PEG2K-PHis26	148.0	149.10 ± 0.989	0.221	0.214 ± 0.012	−11.3	−5.24 ± 4.333
	148.9		0.225		−2.98	
	150.4		0.197		−1.43	
PEG2K-PHis32	207.4	201.90 ± 2.333	0.278	0.278 ± 0.0009	0.29	0.47 ± 0.344
	206.0		0.278		0.18	
	201.9		0.280		0.96	
PEG2K-PHis20 + DOX	179.2	172.53 ± 5.275	0.207	0.233 ± 0.0186	0.19	0.23 ± 0.068
	172.1		0.248		0.18	
	166.3		0.245		0.33	
PEG2K-PHis26 + DOX	226.4	219.36 ± 5.247	0.177	0.207 ± 0.0293	0.21	0.33 ± 0.360
	217.9		0.198		0.81	
	213.8		0.247		−0.05	
PEG2K-PHis32 + DOX	275.8	290.00 ± 10.05	0.299	0.276 ± 0.0308	0.84	0.6 ± 0.171
	297.7		0.233		0.53	
	296.5		0.298		0.44	

Abbreviations: D_h *, Hydrodynamic diameter, DLS *, Dynamic Light Scattering; SD *, Standard Deviation; PDI *, Polydispersity Index, ζ potential *, Zeta Potential.

S5. Doxorubicin Calibration Curves at Different Values of pH by Fluorescence and the Percentages of Dox Released at Different pH Values

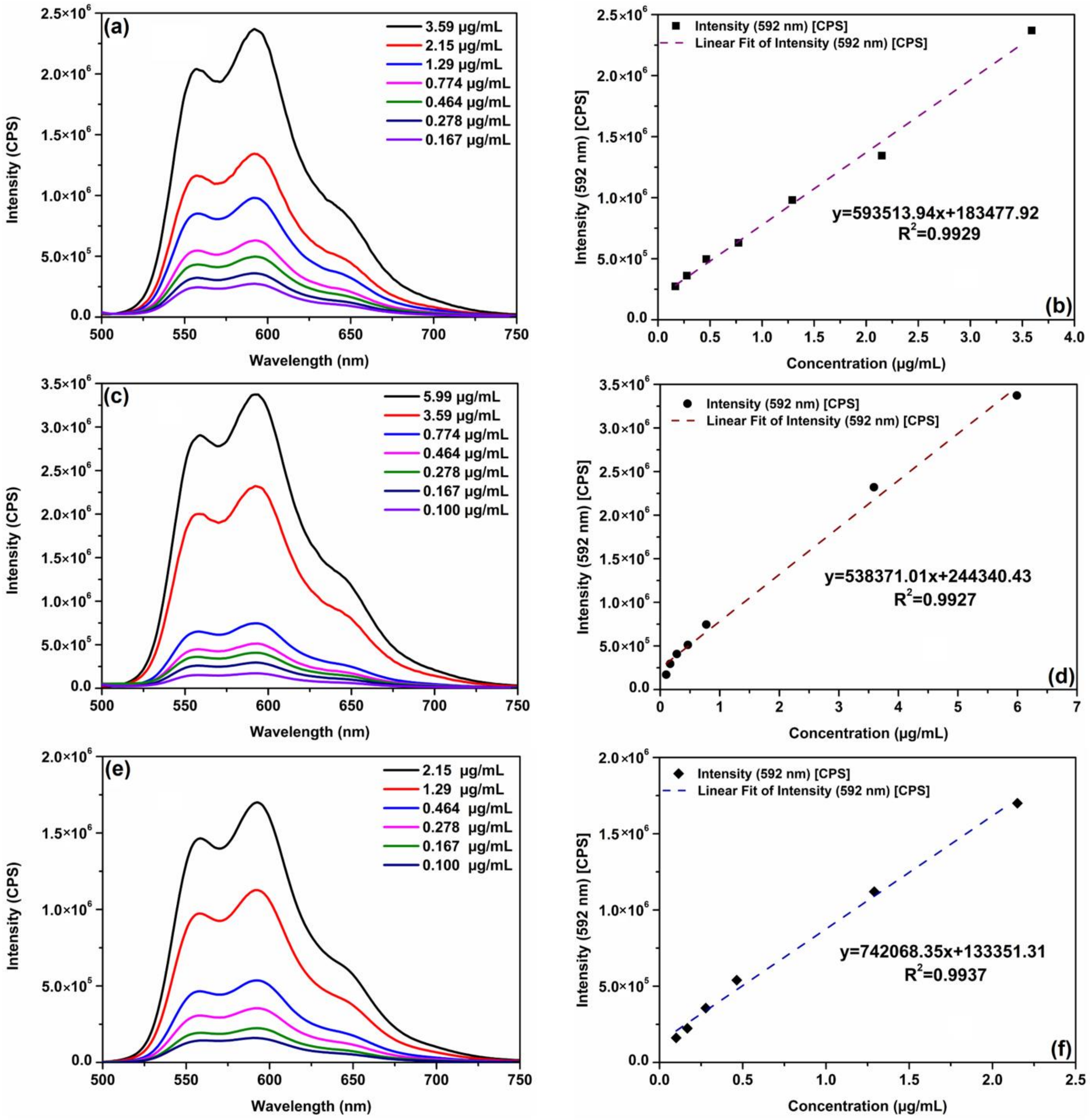

Figure S6. Free DOX calibration curve in 1X PBS at pH 7.4 (**a**,**b**), pH 7.2 (**c**,**d**) respectively pH 6.5 (**e**) and (**f**) by fluorescence with DOX emission peak at 592 nm.

Table S2. The average percentages of DOX release from micelles concomitantly with Free DOX at different times and three pH values of 1X PBS at a temperature of 37 °C. Release studies were performed in triplicate.

Sample Name	Time (h)	pH (% Released ± Standard Deviation %)		
		**7.4**	**7.2**	**6.5**
PEG2K-PHis20 + DOX	0.5	2.91 ± 0.4242	2.24 ± 1.1575	7.14 ± 0.6480
	1	8.61 ± 0.8831	9.02 ± 1.1575	16.56 ± 1.3638
	2	12.89 ± 1.3928	18.23 ± 2.5729	32.08 ± 2.4779
	4	23.71 ± 1.6309	30.92 ± 0.3741	50.32 ± 2.9427
	6	33.15 ± 1.9595	35.30 ± 1.3928	59.93 ± 4.1012
	8	36.23 ± 3.8288	41.25 ± 2.3366	63.34 ± 5.4790
	10	38.88 ± 0.2449	43.13 ± 3.5185	62.48 ± 2.8390
PEG2K-PHis26 + DOX	0.5	3.90 ± 0.7348	2.39 ± 1.1045	4.08 ± 0.4877
	1	5.29 ± 1.8547	11.57 ± 1.3928	11.28 ± 0.4113
	2	14.13 ± 4.0472	20.19 ± 1.7146	27.28 ± 2.8059
	4	26.55 ± 3.2403	36.07 ± 1.9026	45.35 ± 0.9585
	6	32.94 ± 3.4292	41.04 ± 3.5327	53.73 ± 1.1023
	8	35.04 ± 3.9420	45.62 ± 4.4654	57.76 ± 2.2443
	10	36.83 ± 2.6191	46.01 ± 1.5748	56.06 ± 2.5911
PEG2K-PHis32 + DOX	0.5	3.22 ± 0.5099	1.69 ± 0.5099	3.29 ± 0.2484
	1	7.43 ± 1.2083	6.16 ± 2.3790	10.34 ± 0.5768
	2	10.25 ± 1.3490	14.15 ± 3.1591	25.79 ± 1.9266
	4	19.38 ± 0.8831	27.11 ± 3.6359	46.09 ± 2.1191
	6	25.26 ± 1.7146	32.94 ± 2.7604	55.32 ± 1.6064
	8	27.21 ± 2.1354	37.52 ± 1.2569	62.11 ± 2.5575
	10	29.83 ± 1.7204	38.84 ± 0.9899	62.00 ± 3.2119
Free DOX	0.5	8.00 ± 0.7071	11.71 ± 0.9899	7.62 ± 0.7085
	1	19.44 ± 0.8485	41.54 ± 3.9899	16.27 ± 1.0239
	2	30.71 ± 3.5440	57.78 ± 3.9420	30.42 ± 1.5548
	4	48.58 ± 4.0422	79.63 ± 1.6309	48.04 ± 0.9894
	6	55.15 ± 3.5693	92.98 ± 5.8804	55.30 ± 1.1688
	8	59.03 ± 3.2619	98.28 ± 1.5297	56.71 ± 0.9174
	10	62.89 ± 0.6503	98.95 ± 5.5118	52.97 ± 2.8575

S6. Representative Immunofluorescence Images for the Three Types of Unloaded Micelles

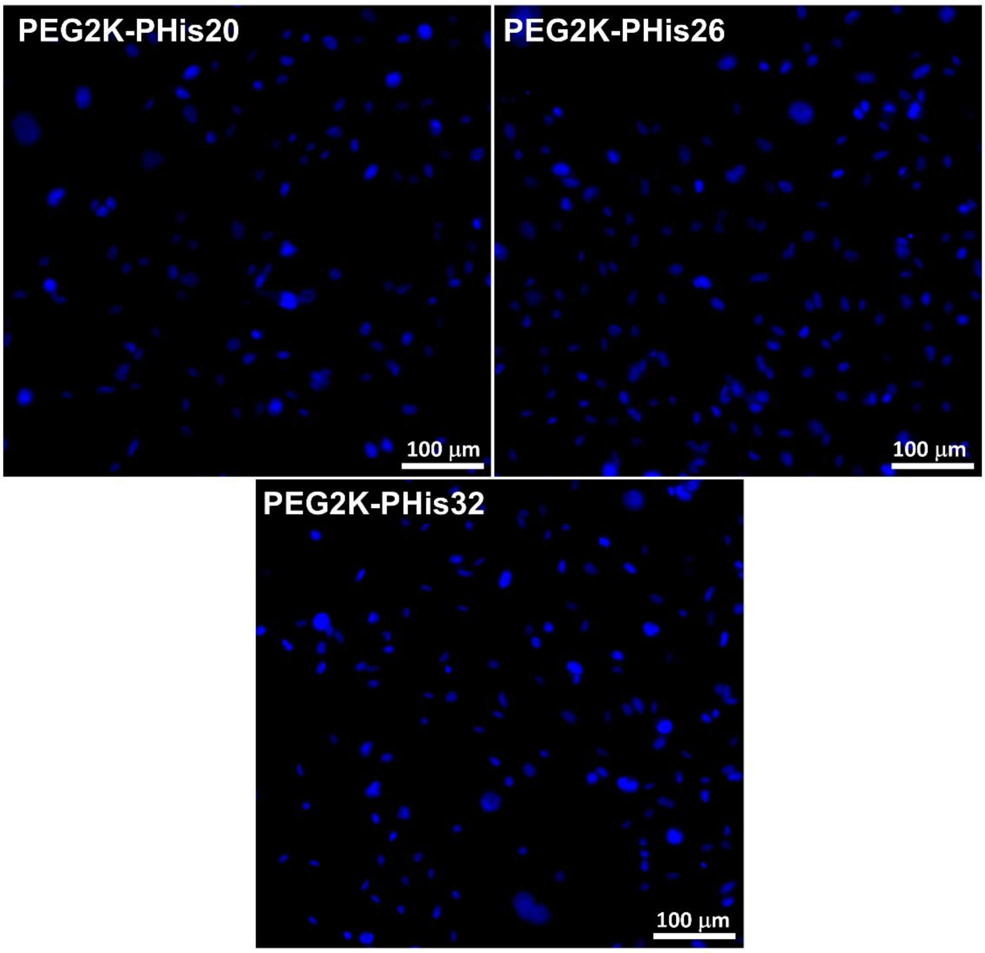

Figure S7. Representative immunofluorescence images for the three types of unloaded micelles, where it can be seen that these micelles do not show fluorescence, and fluorescence is given only by the nuclei of cells stained with NucBlue (Invitrogen).
